# Measuring Mental Effort in Real Time Using Pupillometry

**DOI:** 10.3390/jemr18060070

**Published:** 2025-11-24

**Authors:** Gavindya Jayawardena, Yasith Jayawardana, Jacek Gwizdka

**Affiliations:** 1 IX Lab, School of Information, University of Texas at Austin, Austin, TX 78701, USA; gavindya@utexas.edu; 2 Center for AI in Science and Engineering (ARTISAN), Georgia Institute of Technology, Atlanta, GA 30308, USA; yasith@gatech.edu; 3 Institute of Applied Computer Science, Łódź University of Technology, 90-537 Łódź, Poland

**Keywords:** cognitive load, mental effort, pupillometry, eye-tracking

## Abstract

Mental effort, a critical factor influencing task performance, is often difficult to measure accurately and efficiently. Pupil diameter has emerged as a reliable, real-time indicator of mental effort. This study introduces RIPA2, an enhanced pupillometric index for real-time mental effort assessment. Building on the original RIPA method, RIPA2 incorporates refined Savitzky–Golay filter parameters to better isolate pupil diameter fluctuations within biologically relevant frequency bands linked to cognitive load. We validated RIPA2 across two distinct tasks: a structured N-back memory task and a naturalistic information search task involving fact-checking and decision-making scenarios. Our findings show that RIPA2 reliably tracks variations in mental effort, demonstrating improved sensitivity and consistency over the original RIPA and strong alignment with the established offline measures of pupil-based cognitive load indices, such as LHIPA. Notably, RIPA2 captured increased mental effort at higher N-back levels and successfully distinguished greater effort during decision-making tasks compared to fact-checking tasks, highlighting its applicability to real-world cognitive demands. These findings suggest that RIPA2 provides a robust, continuous, and low-latency method for assessing mental effort. It holds strong potential for broader use in educational settings, medical environments, workplaces, and adaptive user interfaces, facilitating objective monitoring of mental effort beyond laboratory conditions.

## 1. Introduction

The ability to sense a person’s mental effort as it happens could transform how we learn, teach, design software, and operate complex systems. A classroom that adapts lessons to a student’s focus, an interface that simplifies itself before a user becomes overloaded, or a control system that detects cognitive bottlenecks before they lead to error, all depend on measuring mental effort with speed, accuracy, and minimal intrusion. Yet most existing approaches fail to meet all three requirements. Traditional methods, such as self-reports, task accuracy, and reaction-time measures, offer only post-hoc, subjective insights, which makes them unsuited for applications that need continuous, real-time adaptation. While dual-task performance measures such as reaction time and error rates can provide moment-to-moment estimates of cognitive demand, they are often intrusive, disruptive to the primary task, and difficult to deploy continuously in naturalistic settings. These limitations have motivated increasing interest in physiological measures, particularly pupillometry.

Beyond the well-known pupillary light reflex, the pupil dilates transiently during cognitively demanding tasks, a phenomenon known as task-evoked pupillary response (TEPR) [1]. TEPR reflects a time-domain signal, where pupil diameter changes are analyzed over time and aligned to specific stimuli, with baseline adjustments to account for pre-stimulus conditions. TEPR scales with processing demands across memory, language, control, and decision tasks [1,2,3,4,5,6].

In parallel, several algorithms have been developed to analyze pupil diameter in the frequency domain, aiming to estimate mental effort. Notable examples include the Index of Cognitive Activity (ICA) [7,8], the Index of Pupillary Activity (IPA) [9], and the Low–High Index of Pupillary Activity (LHIPA) [10]. These methods detect rapid pupil dilations associated with cognitive load while minimizing the influence of luminance-related changes. ICA is a patented algorithm, while IPA builds upon ICA but offers a more transparent and open-source methodology. LHIPA further refines IPA by providing a more accurate separation between cognitively driven and luminance-induced fluctuations. Despite their advancements, all three algorithms are limited by their offline nature, restricting their applicability in real-time settings.

To overcome this, Jayawardena et al. [11] developed the Real-Time Index of Pupillary Activity (RIPA), an extension of LHIPA designed for near-real-time cognitive load estimation. RIPA uses two parallel Savitzky–Golay (SG) filters [12,13,14] with different parameters to compute first-derivative estimates of the pupil signal, from which it calculates a very low frequency (VLF) to low frequency (LF) ratio and peak rate. This approach enhances sensitivity to cognitively driven fluctuations while reducing luminance effects.

Unlike traditional spectral methods, RIPA’s use of SG filters is not explicitly designed to isolate specific frequency bands. Instead, the filters are chosen to enhance responsiveness for real-time signal tracking, prioritizing temporal dynamics over spectral accuracy. However, the current filter settings in RIPA do not align well with the frequency range most associated with mental effort, typically below 4 Hz [15,16,17,18,19], highlighting an opportunity to better incorporate spectral characteristics into the algorithm’s design. Furthermore, RIPA has yet to be evaluated in more naturalistic task settings, leaving its performance in real-world applications an open question.

In this paper, we make two primary contributions: First, we make key improvements to the RIPA algorithm based on prior research in frequency-domain pupillary analysis to better capture the physiological dynamics underlying mental effort. We name this improved algorithm RIPA2. By selecting SG filter lengths and polynomial orders according to the sampling rate, and using a simpler point-wise metric, RIPA2 more accurately isolates the VLF and LF components linked to mental effort. This modification enhances the reliability of the cognitive load estimate while maintaining responsiveness, making RIPA2 more suitable for real-time applications.

Second, we extend the applicability of RIPA2 by evaluating it in a task domain where the original RIPA had not previously been tested: a naturalistic information-search task involving fact-checking (FC) and decision-making (DM). We evaluate RIPA2 in two different task paradigms:

1. **N-back task**—A standard benchmark for tracking mental effort across graded difficulty. It is a structured cognitive task in which participants must indicate whether the current stimulus matches the one they observed *n* steps earlier.
2. **Information-Search task designed for FC and DM**—A more naturalistic task designed to capture the complexities of real-world cognitive demands.

The remainder of this paper is organized as follows: We begin by presenting relevant background, followed by a detailed description of the RIPA2 implementation. Next, we report the results from two experimental tasks, accompanied by a brief discussion of the key findings. Finally, we provide a general discussion, summarizing the main contributions and outlining directions for future research.

## 2. Background

Cognitive load theory provides a foundational framework for understanding how mental effort is distributed during learning and task performance by emphasizing that working memory has a limited capacity [20,21]. It differentiates between mental demands arising from the complexity of the task, those shaped by how information is presented, and those associated with how learners allocate their cognitive resources [20,22,23]. In applied domains such as interface design and workload assessment, cognitive load is often inferred from physiological measures, with pupillometry offering a non-invasive indicator of mental effort [1,24].

Prior research has employed both time-domain and frequency-domain analyses of pupil diameter to track cognitive load, showing that pupil dilation reliably increases under higher cognitive demand  [1,2,25]. These studies have further linked pupil dynamics to attentional processes, evidence accumulation, and listening effort, demonstrating that both the magnitude and rate of pupil dilation can predict performance accuracy and efficiency in cognitively demanding tasks  [1,26,27]. Beyond reflecting ongoing cognitive load, pupil dilation has also been proposed as an index anticipatory effort, as evidenced by larger pupil sizes during the preparatory phase of anti-saccade tasks  [28], and prior to stimulus onset in intermixed pro/anti trial designs [29], reflecting elevated preparatory control demands

While much of this research has focused on the magnitude and timing of pupil dilation, growing interest has been directed toward the frequency characteristics of pupil signals, particularly those associated with cognitive processing [30,31]. In pupillometry, such cognitively meaningful fluctuations typically occur below 4 Hz [15], whereas higher frequencies are generally attributed to noise or measurement artifacts rather than physiological relevance. Prior studies [16,17,18,19] recommend a 4 Hz low-pass filter to preserve task-relevant pupil dynamics while minimizing high-frequency noise. Frequency-domain research reinforces this focus on lower frequency ranges. Nakayama and Shimizu [32] observed increased power spectral density (PSD) in the 0.1–0.5 Hz and 1.6–3.5 Hz bands during arithmetic tasks. Peysakhovich et al. [15] found elevated PSD under working memory load in the 0–4 Hz range compared to a control condition. They redefined the frequency bands to span 0–1.6 Hz for VLF and 1.6–4 Hz for LF, demonstrating that these ranges are sensitive to cognitive load while remaining largely unaffected by ambient lighting changes. More recently, Medeiros et al. [33] identified an optimal frequency band combination for measuring VLF and LF components of pupil diameter. In a controlled study with 21 programmers performing software bug inspection tasks, they found that the 0.06–0.29 Hz band was optimal for VLF, while 0.29–0.49 Hz was best for LF. These bands showed strong correlations with task-induced cognitive load, aligning well with physiological expectations.

Building on this frequency-domain perspective, several computational methods have been developed to estimate mental effort from pupil dynamics, with one of the earliest and most influential being the ICA [7,8]. ICA detects rapid pupil dilations linked to cognitive load. It is a patented algorithm that incorporates several pre-processing steps to handle blinks, wavelet decomposition to capture changes in pupil dilation, and thresholding to denoise the signal.

IPA [9] builds on the ICA algorithm but introduces a transparent methodology and improves robustness by reducing sensitivity to luminance-related pupillary changes. It starts by excluding pupil signals during blinks using a 200 ms window. The calculation involves a two-level Symlet-16 wavelet decomposition of the pupil dilation signal, followed by multi-resolution analysis to smooth the signal. From the resulting decomposition, a level is arbitrarily chosen to produce a smoother approximation of the signal. The next step involves thresholding the wavelet modulus maxima coefficients using a universal threshold [9]. The remaining coefficients after thresholding contribute to the IPA reading, providing a measure of pupil diameter fluctuations indicative of cognitive activity.

LHIPA [10] refines the IPA by using wavelet decomposition to separate luminance-driven VLF changes from cognitively driven LF changes in pupil size. Instead of thresholded coefficients, LHIPA counts modulus maxima in wavelet bands. The wavelet analysis involves decomposing the pupil diameter signal into LF and VLF components at multiple resolution levels. The ratio of VLF to LF wavelet coefficients provides the LHIPA measure. As cognitive load increases, pupil dilation also increases, resulting in a decrease in the LHIPA ratio due to higher amplitude in LF components relative to VLF ones.

Since ICA, IPA, and LHIPA share the limitation of being offline algorithms, Jayawardena et al. [11] introduced RIPA, an approach designed for near-real-time cognitive load estimation, inspired by LHIPA. RIPA mimics the behavior of LHIPA by replacing wavelet decomposition with two Savitzky–Golay (SG) first-order derivative filters [12,13,14] to approximate the VLF-to-LF ratio used in LHIPA. Unlike standard smoothing filters, SG derivative filters extract local rate-of-change features such as onsets and rapid transitions by combining smoothing and derivative estimation via polynomial least-squares fitting. This makes them well-suited for real-time pupil signal analysis. By applying these filters at different smoothing levels, RIPA differentiates the pupil signal to isolate task-evoked responses from luminance-driven fluctuations. This process reduces high-frequency noise, increases sensitivity to cognitively driven changes, and minimizes luminance effects.

Despite its advantages, RIPA does not explicitly target frequency bands known to be associated with cognitive processes. Instead, its use of SG filters prioritizes responsiveness and temporal resolution over spectral precision. This highlights an opportunity to refine RIPA’s design by incorporating more targeted spectral considerations, potentially improving its sensitivity to mental effort.

## 3. Methodology

First, we describe the original RIPA algorithm and point out its limitations. Then, we present the development of RIPA2, an enhanced variation of the original RIPA algorithm [11], incorporating key improvements informed by prior research in frequency-domain pupillary analysis. In particular, we explain the optimization of SG derivative filter parameters to improve the algorithm’s sensitivity and robustness.

### 3.1. RIPA Algorithm

In RIPA, two first-order SG derivative filters are applied to separate frequency components, thereby approximating the VLF-to-LF ratio [11]. Each SG filter consists of 2M+1 coefficients defined by three parameters: half-width *M*, polynomial order *N*, and derivative order *D* (fixed at D=1 in RIPA). Since these coefficients are independent of the input data, they can be pre-computed. The filtering operation is mathematically equivalent to sliding a window of 2M+1 samples across the signal, fitting an *N*-th degree polynomial to each window, and computing its *D*-th derivative at the window center.

In RIPA, the first SG filter uses M=6, N=2, and the second uses M=6, N=10. Their corresponding normalized cutoff frequencies are $f_{c}\approx 0.165$ and $f_{c}\approx 0.681$, respectively, with both filters attenuating components above their cutoff. These $f_{c}$ are taken from Schafer  [12], who computed impulse responses for various combinations of *M* and *N* providing $f_{c}$ for filters with M=2,3,4,5,6 and N=2,4,6,8,10. Based on these data, the *M* and *N* values were selected to produce two low-pass responses for the SG filters, which are used to compute the derivatives of pupil diameter at the VLF and LF cutoff frequencies. This configuration enables computation of the VLF/LF ratio in a manner analogous to the LHIPA computation, but at a substantially reduced computational cost. The normalized cutoff frequency $f_{c}$ is discussed in detail later in the text.

Real-time processing in RIPA relies on multiple double-ended buffers that store raw and filtered signals, frequency components, their ratio, and derived features for continuous analysis. To identify significant oscillations in the VLF/LF ratio, RIPA computes modulus maxima while preserving the signal’s sign. A threshold based on the standard deviation of these maxima is then applied. RIPA quantifies mental effort by counting spikes where the VLF/LF ratio exceeds this threshold. Finally, to make the measure intuitive and comparable across individuals, RIPA is normalized and inverted, such that higher values correspond to increased cognitive load.

The empirical validation of the RIPA algorithm was conducted using data sampled at 1000 Hz [11]. The normalized cutoff frequencies are expressed as the ratio of the target cutoff frequency to the Nyquist frequency: $f_{c}=\frac{f_{\mathrm{target}}}{f_{\mathrm{Nyquist}}}$. With a sampling rate of 1000 Hz, the Nyquist frequency is 500 Hz. Applying this equation to the normalized cutoff frequency values reported in RIPA, $f_{c}\approx 0.165$ and $f_{c}\approx 0.681$, corresponds to absolute cutoff frequencies of $f_{\mathrm{target}}=0.165\times 500=82.5\;\mathrm{Hz}$ and $f_{\mathrm{target}}=0.681\times 500=340.5\;\mathrm{Hz}$, respectively. However, these values exceed the biologically meaningful frequency range in pupillometry, where cognitively driven fluctuations typically occur below 4 Hz [15,16,17,18,19,32,33]. This indicates that RIPA’s current filter settings do not align with the frequency bands most associated with mental effort. Therefore, we modified the algorithm by incorporating spectral considerations into its design.

### 3.2. Development of RIPA2

Our goal was to match the SG filters’ frequency responses to physiologically meaningful bands identified in prior work. For the first SG filter, which targets the VLF component, we set a target cutoff frequency of ≈0.29 Hz [33]. For the second SG filter, targeting the LF component, we aimed for a cutoff near 4 Hz to capture cognitively evoked fluctuations, aligning with frequencies reported in previous studies [15,16,17,18,19,32].

We used pupillometry data sampled at 300 Hz and selected parameters *M* and *N* for the two derivative SG filters based on Schäfer’s linear model for estimating the normalized cutoff frequency $f_{c}$ [12]. Schäfer’s approximation, valid for longer SG filters where M≥25 and N<M, is given by

$$f_{c}=\frac{N+1}{3.2M-4.6}$$

This approximation provides a useful guideline for selecting SG filter parameters to isolate physiologically relevant frequency ranges.

Given the 300 Hz sampling rate of the collected pupillometry data, the corresponding Nyquist frequency is 150 Hz. Using the equation $f_{c}=\frac{f_{\mathrm{target}}}{f_{\mathrm{Nyquist}}}$, a target cutoff of 0.29 Hz yields $f_{c}\approx 0.00193$, and a target of 4 Hz yields $f_{c}\approx 0.0267$. Based on these normalized values, we selected SG filter parameters *M* and *N* to approximate the corresponding VLF and LF components.

While shorter filters often enable better real-time responsiveness [12], we prioritized physiological accuracy by balancing processing speed with precise frequency selectivity in our parameter choices. To capture very slow changes at 0.29 Hz, we used a large window size (973 points, $M_{VLF}=486$) with a low polynomial order ($N_{VLF}=2$). This configuration ensures that the derivative estimate predominantly reflects VLF components. For extracting rapid, effort-related dynamics at 4 Hz, we used a smaller window (121 points, $M_{LF}=60$) and a higher polynomial order ($N_{LF}=4$). This enables the filter to track faster signal variations while maintaining sufficient noise suppression. These parameter choices were guided by evaluating the frequency response of each SG derivative filter to ensure accurate signal differentiation below the target frequencies and robustness in real-time applications.

Based on our parameter selection, the resulting SG first-order derivative filters are as follows:

- ${\mathrm{SG}}_{\mathrm{VLF}}$: Targets cutoff frequency ≈0.29 Hz ($f_{c}\approx 0.00193$), using a window size of 973 points ($M_{VLF}=486$) and polynomial order $N_{VLF}=2$, to isolate slow, luminance-related trends.
- ${\mathrm{SG}}_{\mathrm{LF}}$: Targets cutoff frequency ≈4 Hz ($f_{c}\approx 0.0267$), using a window size of 121 points ($M_{LF}=60$) and polynomial order $N_{LF}=4$, to extract fast, effort- related changes.

Using $SG_{VLF}$ and $SG_{LF}$, we simultaneously computed two derivatives of the pupil diameter signal, capturing both VLF and LF fluctuations in pupil dynamics in near-real time. To support this, we maintained a rolling buffer of pupil diameter samples using a Python 3.11 deque data structure, consistent with the original RIPA algorithm. The buffer size was set to 1200 samples, corresponding to 4 s of data at a 300 Hz sampling rate. For this sampling rate, the chosen window length was sufficient to accommodate the $SG_{VLF}$ filter (973 points) while still allowing the shorter SG filter (121 points for $SG_{LF}$) to operate effectively. A shorter window would not provide enough data for the VLF filter to capture slow, physiologically meaningful trends.

Based on the filtered responses VLF and LF, we computed the RIPA2 value as the difference of squared LF and VLF components of the pupil signal, P[t]:

$$SG_{LF}=f\left(M_{LF},N_{LF}\right)\;SG_{VLF}=f\left(M_{VLF},N_{VLF}\right)$$

$$RIPA2\left[t_{i}\right]={\left(SG_{LF}\cdot P\left[t_{i}\pm M_{LF}\right]\right)}^{2}-{\left(SG_{VLF}\cdot P\left[t_{i}\pm M_{VLF}\right]\right)}^{2}$$

Here, the parameters of the SG first-order derivative filters are expressed as functions of their half window length and polynomial order. Each filter is applied over its corresponding data window $P[t_{i}\pm M_{LF}]$ and $P[t_{i}\pm M_{VLF}]$, reflecting their designed filter widths. At each time point $t_{i}$, we analyzed a 1200-sample segment of the pupil diameter signal. RIPA2 computation begins once the buffer holds enough data to cover the largest filter window (VLF), ensuring sufficient context for accurate differentiation. After this, the filters advance with each new sample, enabling continuous, near-real-time computation.

Because the VLF and LF filters use different window sizes, their outputs are not temporally aligned. To compensate for this discrepancy, we adjusted the indexing so that both filters’ outputs corresponded to the same effective center time. Specifically, we align the LF output by applying an offset equal to the difference in filter delays: $\Delta =M_{VLF}-M_{LF}$.

In addition to modifying the SG filter parameters, RIPA2 differs from the original RIPA by using the difference of the squared LF and VLF components instead of their ratio. The larger SG filter window sizes employed here generated smoother LF and VLF signals with fewer transients. This allowed us to use a point-wise metric that captures the relative dominance of LF activity with respect to VLF activity, yielding RIPA2 values which characterize the extent of fast and slow fluctuations in pupil dynamics. Higher values indicate increased LF activity, often associated with elevated mental effort, while lower values suggest smoother, luminance-driven trends. The following pseudo-code (Algorithm 1) shows the computation of RIPA2.
**Algorithm 1** Computation of RIPA2 Values from SG-Derivative Filtered Signals  1:let i = 0  2:**function** Ripa2Function(P)  3:  i ← i + 1  4:  value_VLF_←ApplySGFilter(SG_VLF_, P[$t_{i}$ ± M_VLF_])  5:  value_LF_←ApplySGFilter(SG_LF_, P[$t_{i}$ ± M_LF_])  6:  **if** i $\geq 2M_{\mathrm{VLF}}+1$ **then**  7:    ripa2 ← (value_LF_)^2^ − (value_VLF_)^2^  8:    ripa2 ← Clip(ripa2, 0.0, 1.5)  9:    Append(list_RIPA_, ripa2)10:  **end if**11:**end function**

**Figure 1:**
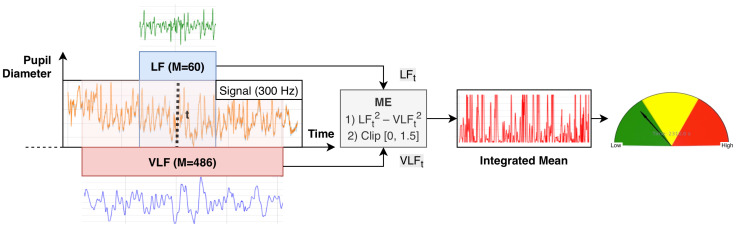
This figure illustrates the processing of pupil diameter measurements through two low-pass SG derivative filters. The orange signal represents the raw pupil-diameter signal. The green signal shows the output after filtering with $SG_{LF}$, and the blue signal shows the output after filtering with $SG_{VLF}$. These filtered signals are used to compute the RIPA2 metric, defined as the difference between the squared LF and VLF components. The resulting RIPA2 value is then clipped to the range 0–1.5, and the final clipped signal is shown in red.

To ensure numerical stability and facilitate downstream processing and real-time visualization, the RIPA2 output was clipped to the fixed range of [0, 1.5]. This limited the influence of outliers and made the metric more interpretable. Within this range, values near 0 reflect low mental effort, while values near 1.5 indicate heightened mental effort. Figure 1 illustrates the overall processing pipeline that transforms pupil-diameter measurements into the RIPA2 signal.

### 3.3. Smoothed RIPA2 Signal

**Figure 2:**
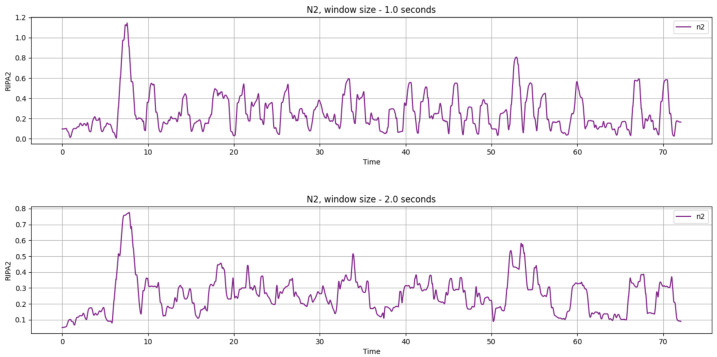
RIPA2 smoothed signal with 1 s (top) and 2 s (bottom) window sizes; the x-axis denotes time in seconds.

We applied smoothing to support the mental-effort gauge visualization. Since RIPA2 is computed for each pupil diameter sample, we further processed the signal by calculating its moving average over a configurable time window. This was achieved by convolving the RIPA2 time series with a simple moving average filter, which smooths the signal, reduces noise, and emphasizes longer-term trends in cognitive load. The window size is customizable, allowing for flexibility in smoothing level, such as 1 s or 2 s (see Figure 2).

We used a 1–2 s moving-average windows because it matches the fast, phasic timescales of pupil responses driven by neuromodulators. Noradrenergic activity produces dilations within 0.8–1.1 s after cognitive events [34], and orexin activation similarly causes rapid, causal increases in pupil size [35]. Together, these findings support these windows as optimal for capturing quick cognitive-load fluctuations while still smoothing noise.

### 3.4. Mental Effort Gauge Visualization

**Figure 3:**
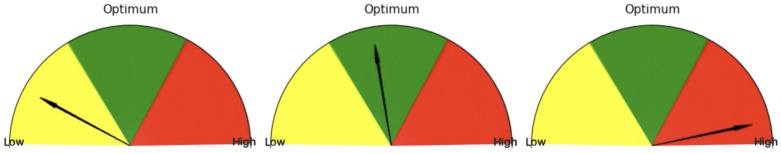
This figure shows snapshots of the near-real-time visualization of mental effort, represented as a gauge, using the RIPA2 signal. The gauge dynamically updates to reflect mental effort, with the signal smoothed over a moving window.

Using the smoothed RIPA2 signal, we created a dynamic animation that visualizes mental effort as a gauge in real-time. For the real-time visualization, we generated a series of frames by selecting values from the smoothed signal at regular intervals. These frames were used to update a polar plot, as shown in Figure 3, where mental effort is represented as a gauge on a circular axis. The plot was updated continuously, providing a real-time approximation of mental effort changes over time. Such a gauge could be integrated into future adaptive interfaces to support real-time cognitive load monitoring and context- aware feedback.

## 4. Evaluation of RIPA2

To validate RIPA2 and demonstrate its utility, we conducted an eye-tracking experiment using a within-subjects design across two distinct task paradigms. The first task focused on validating RIPA2’s sensitivity to varying levels of mental effort induced by task difficulty. For this, we employed the N-back task, a well-established benchmark for evaluating cognitive load across multiple levels of difficulty. This task allowed us to test whether RIPA2 could effectively track fluctuations in mental effort as the cognitive demands of the task increased.

The second task showcased the applied utility of RIPA2 in capturing the dynamics of mental effort during a more naturalistic scenario. Participants engaged in an information-search task involving fact-checking (FC) and decision-making (DM), designed to reflect the complex cognitive demands of real-world decision-making and information retrieval processes.

### 4.1. Participants

We conducted our recruitment through emails and posted flyers around campus. Interested individuals completed a pre-screening questionnaire to assess eligibility. To qualify, participants needed to be fluent in English, aged 18–60, and able to sit comfortably at a computer monitor placed 55–70 cm away. For those wearing corrective lenses, only single-focus lenses with a vertical height of at least 15 mm were included. From those who expressed interest, we recruited 30 participants (13 male, 17 female; ages ranging from 18 to 37 years (M = 24.2, SD = 4.74). All the participants had normal or corrected-to-normal vision. The participants were compensated with a USD 30 Amazon gift card upon successful completion of the study.

### 4.2. Apparatus

Both tasks were conducted in a quiet, laboratory environment using the Tobii TX300 screen-based eye-tracker (manufacturer-reported accuracy: 0.4–0.9°; precision: 0.04–0.15°), which recorded gaze data at a sampling rate of 300 Hz, in combination with the iMotions software running on a desktop Windows computer. The tasks were implemented as two separate studies within iMotions: one for the N-back task and another for the information search task, facilitating a smooth transition between tasks. Stimuli were presented on a 24-inch monitor with a resolution of 1920 × 1080 pixels. A standard keyboard and mouse were used for participant responses.

The physical setup was carefully adjusted to optimize data quality. The participants were seated at a distance of 57 to 65 cm from the monitor, with their seating height and head position adjusted to align their eyes with the eye-tracker’s infrared field. The eye-tracker’s angle and position were also adjusted to ensure continuous visibility of both eyes during calibration and task performance.

### 4.3. Procedure

Upon arrival, the participants read and signed the informed consent form, and then completed a brief questionnaire assessing their familiarity with three topics: electronic devices, travel destination cities, and diets. This information was used to select three DM scenarios for each participant during the information search task, with the questions chosen based on the areas in which they reported the least familiarity.

The participants were then seated at the eye-tracking station and provided with verbal instructions outlining the study flow. They first completed the N-back task, which began with a training phase covering all difficulty levels. Upon successful completion of the training, the eye-tracker was calibrated using the 5-point calibration method in iMotions. The participants then proceeded with the main N-back task, which lasted approximately 30 min.

After completing the N-back task, the participants took a five-minute break, after which a second 5-point eye-tracking calibration was conducted in preparation for the information search task. This task began with the DM scenarios, followed by the FC tasks, lasting approximately 60 min in total.

### 4.4. Data Analysis

During both tasks, eye-tracking data were continuously recorded along with behavioral responses. Raw pupil diameter data were accessed through the iMotions API, which supports real-time replay of the data stream. This replay capability was leveraged to simulate a near-real-time processing pipeline for testing the RIPA2 algorithm.

#### 4.4.1. Artifact Removal and Data Selection

Blink artifacts and other discontinuities in the pupil diameter signal were identified using markers produced by the eye-tracking software. Invalid pupil diameter values were explicitly labeled as −1, and we removed these values along with 100 ms (30 samples) before and after each blink event to eliminate pre- and post-blink distortions, such as anticipatory pupil constriction or recovery dilation, following methods similar to those in other studies [10,33]. These removed intervals are referred to as masked sections.

Two strategies for handling these gaps were considered:

1. Gap removal: Completely dropping the masked sections from the time series.
2. Gap interpolation: Reconstructing missing values using an auto-regressive model fitted to the surrounding data.

For this study, we adopted the gap removal approach in order to preserve the temporal integrity of a real-time signal processing system. Interpolation-based techniques were excluded from our implementation to avoid introducing forward-looking bias. Although blink rate and blink patterns are known to correlate with cognitive load [36,37], RIPA2 is designed to quantify rapid fluctuations in pupil diameter, and thus blink events are treated as artifacts rather than informative features.

#### 4.4.2. Computation of Mental Effort Estimators

We implemented the pre-processing and computation of mental effort estimators using Python. In addition to RIPA2, we computed LHIPA and original RIPA as baseline measures. These three indices were used as dependent variables in our analysis.

- LHIPA: We computed LHIPA values directly from the pupil diameter signals using the algorithm implemented in Python based on the version provided by Duchowski et al. [10]. For the discrete wavelet transform, we selected the Daubechies wavelet of order 16, which consists of 32 coefficients, suitable for data sampled at 300 Hz. This choice incorporates a sampling window of 128 ms (32 × 4), as recommended.
- RIPA: We computed RIPA signals using the original RIPA algorithm [11]. The only adjustment made was to the queue length parameter, which was set to 300 data points, corresponding to one second of data at the 300 Hz sampling frequency used in this study.
- RIPA2: We computed RIPA2 signals as described in the Methodology Section. To reiterate, the RIPA2 algorithm employed two SG derivative filters: (1) a LF filter with parameters $M_{LF}=60$, $N_{LF}=4$ and (2) a VLF filter with parameters $M_{VLF}=486$, $N_{VLF}=2$. A rolling window of 1200 samples was used, corresponding to 4 s of data at the 300 Hz sampling rate. The resulting RIPA2 values ranged from 0 (indicating low mental effort) to 1.5 (indicating high mental effort).

## 5. Task 1: N-Back Task

### 5.1. Method

We selected the N-back task to explore the relationship between task difficulty and active working memory [20], and to assess how RIPA2 captures corresponding changes in mental effort. Previous research by Duchowski et al. [10] and Jayawardena et al. [11] demonstrated the utility of LHIPA and RIPA using the N-back task. Studies have also shown that larger pupil dilations correlate with higher cognitive load during N-back tasks [25,38,39,40].

Our N-back task followed the experimental protocol used by Duchowski et al. [10] and Appel et al. [41], where participants were presented with a sequence of letters drawn from the set L = {C, F, H, S}. In contrast to the two difficulty levels and a baseline task used by Duchowski et al. [10], our N-back task included four difficulty levels (n0, n1, n2, and n3), with N-back level manipulated as an independent variable.

In the N-back task, the participants were required to recall and compare letters from n trials prior. The participants indicated whether the currently shown letter matched the one from n trials before by pressing space bar on the keyboard. Each trial consisted of a 0.5 s stimulus presentation followed by a 1.5 s inter-stimulus interval, resulting in a 2 s trial duration. Levels n0–n2 had 45 trials each, while level n3 had 30 trials. The participants completed 16 sessions in total, four of each N-back level (n0 to n3). The order of sessions was randomized, with no more than two consecutive sessions of the same difficulty.

Similar to Duchowski et al. [10], each letter in our task was presented in one of five randomly selected locations on the screen (center, top-left, top-right, bottom-left, and bottom-right). This spatial randomization served two purposes: (1) to ensure the participants focused on the letter’s identity rather than relying on spatial cues, and (2) to capture pupil diameter across various gaze angles.

Before the main task, the participants underwent training to familiarize themselves with all four difficulty levels. Training continued until they met the performance thresholds: at least 80% accuracy for n0, n1, and n2 levels, and at least 65% accuracy for the more challenging n3 level. This ensured that the participants understood the task and could perform reliably before data collection.

Of the 30 participants, data from two were excluded from the analysis due to technical issues. The analysis was further limited to N-back sessions (excluding training) in which the participants achieved accuracy greater than 80%. We hypothesized that as task difficulty increases (i.e., as N-back level increases), RIPA2 values will also increase, reflecting higher mental effort associated with remembering a larger sequence of items.

### 5.2. Results

For each participant, we computed the LHIPA, original RIPA, and RIPA2 values for the 16 N-back tasks they completed, using these indices as the dependent variables in our analysis. We first examined their descriptive statistics and tested for normality. In cases of deviations from normality, we applied a Box–Cox transformation to normalize the data. Finally, we performed a repeated measures one-way ANOVA to assess the statistical significance of the results.

#### 5.2.1. LHIPA

**Figure 4:**
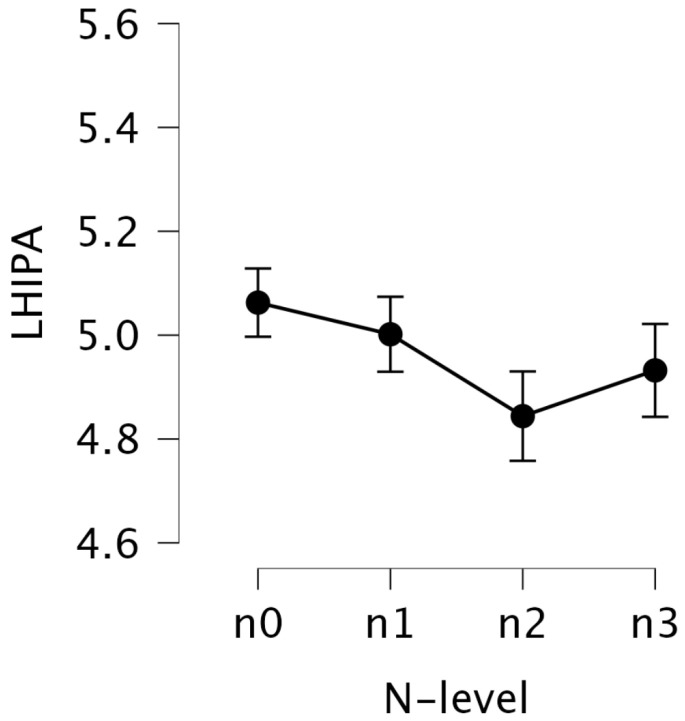
Mean LHIPA values with standard error bars for each N-back level.

Descriptive statistics showed a gradual decrease in the LHIPA values with increasing N-back levels: from M = 5.063 (SD = 0.692) at the n0 level to M = 4.844 (SD = 0.865) at the n2 level (see Figure 4). Given that LHIPA is inversely related to cognitive load, this suggests that the participants experienced higher mental effort during the n1, n2, and n3 levels compared to n0. Interestingly, the n2 level exhibited the lowest LHIPA value, indicating that it was associated with the highest mental effort among all the N-back levels.

Although the trend of the LHIPA values suggested a possible effect of task difficulty, we conducted formal statistical tests to evaluate the significance. Levene’s test for homogeneity of variances across N-levels was non-significant, F(3, 407)=1.90, p=0.129, indicating that the assumption of equal variances was met. Though the Shapiro–Wilk test indicated significant deviations from normality for all the N-back levels (p<0.05), skewness values ranged from −0.769 to −0.419, and kurtosis values ranged from −0.790 to 0.195, suggesting only mild departures from normality (see Table 1).

To correct for violations of normality and variance homogeneity, a Box–Cox transformation (λ=2.473) was applied. The transformed data showed improved normality (Shapiro–Wilk p>0.148 across all levels) and homogeneity of variance (Levene’s test: F(3, 407)=0.533, p=0.661) (see Table 2).

A repeated measures one-way ANOVA on Box–Cox-transformed LHIPA revealed a significant main effect of N-back level, F(3, 75)=4.987, p=0.003, $\eta^{2}=0.018$, indicating a small effect size. $\eta^{2}$ is a common effect size measures, where 0.01 is interpreted as a small effect, 0.06 a medium effect, and 0.14 a large effect [42].

Post hoc paired comparisons using the Benjamini–Hochberg procedure revealed trends toward significance between several N-back levels (see Table 3). Notably, differences were observed between n0 and n2 conditions (p=0.069) and between n1 and n2 conditions (p=0.069), and between n0 and n3 (p=0.054) and n1 and n3 (p=0.054). These results suggest reduced LHIPA at the n2 and n3 levels compared to the lower N-back levels, indicating an increase in mental effort required at these higher difficulty conditions.

Although the *p*-values did not reach the conventional threshold for statistical significance, Bayesian analysis provided moderate evidence supporting true differences for n0 vs. n2 (BF10=1.66), n1 vs. n2 (BF10=1.34), n0 vs. n3 (BF10=3.65), and n1 vs. n3 (BF10=2.90). The effect sizes were small to moderate, with Hedges’ *g* ranging from 0.20 to 0.31 across these contrasts, suggesting subtle but meaningful changes in LHIPA across N-back levels. Hedges’ g quantifies the standardized difference between two group means, with suggested benchmarks indicating a small effect (≈0.2), medium effect (≈0.5), and large effect (≈0.8) [43].

#### 5.2.2. RIPA

**Figure 5:**
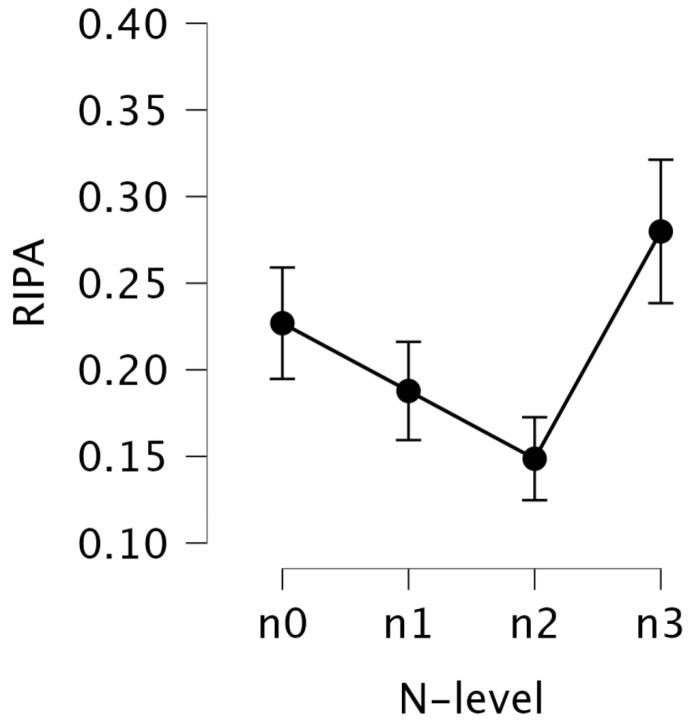
Mean RIPA values with standard error bars for each N-back level.

In contrast to LHIPA, RIPA did not capture the expected effect of task difficulty. While RIPA is positively related to mental effort, it showed the lowest mental effort at the n2 level and the highest at n3 (see Figure 5). Notably, the mental effort at n3 was higher than all the n-levels, but both n1 and n2 showed lower effort compared to n0. This pattern is inconsistent with prior studies [25,38,39,40], which have typically found that cognitive load increases as task difficulty rises.

To investigate this further, we conducted a statistical analysis. Levene’s test revealed a significant violation of the homogeneity of variance assumption for RIPA across N-back levels, F(3, 407)=12.94, p<0.001. Additionally, the raw data exhibited substantial skewness and kurtosis indicating heavy-tailed and asymmetric distributions. These deviations from normality were confirmed by the Shapiro–Wilk test, which was significant for all the N-back levels (p<0.001) (see Table 4).

To address these violations, a Box–Cox transformation was applied (λ=−0.4277), which substantially reduced skewness and kurtosis (skewness range: −0.005 to 0.417; kurtosis range: −0.857 to 0.504), and normalized the distributions across all levels (Shapiro–Wilk p>0.384). Following the transformation, Levene’s test was no longer significant, F(3, 407)=1.012, p=0.391, indicating that the assumption of equal variances had been adequately met (see Table 5).

A repeated measures one-way ANOVA was conducted to assess the effect of N-back level on Box–Cox-transformed RIPA. The results indicated no significant main effect of N-back level, $F\left(3,\;75\right)=0.784,\;p=0.506,\;\eta^{2}=0.019,$ suggesting that changes in task difficulty was not reliably captured by RIPA. The effect size was small. Although the overall effect was non-significant, pairwise post hoc comparisons were performed for completeness using the Benjamini–Hochberg procedure. As shown in the Table 6, none of the contrasts reached statistical significance (all *p*-values ≥0.05 after correction). Moreover, Bayes factors (BF_10_ < 1) provided more support for the null hypothesis than for any real differences between conditions. This suggests that there were no substantial differences in the RIPA values across the N-back levels.

#### 5.2.3. RIPA2

**Figure 6:**
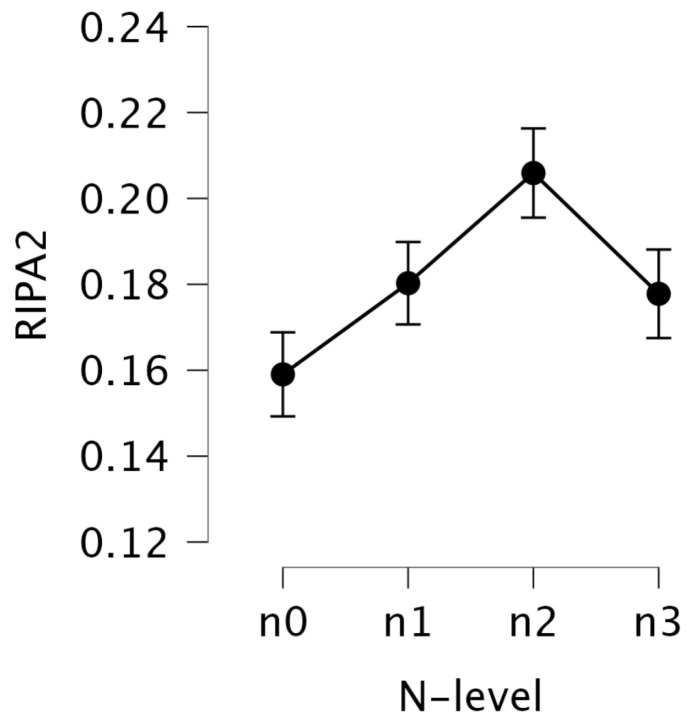
Mean RIPA2 values with standard error bars for each N-back level.

Descriptive statistics for RIPA2 across the N-back levels (n0–n3) are presented in Table 7, with corresponding trends visualized in Figure 6. The mean values of RIPA2 increased from n0 (M = 0.159, SD = 0.103) to n2 (M = 0.206, SD = 0.104), followed by a slight drop at n3 (M = 0.178, SD = 0.096). Since RIPA2 is designed to increase with mental effort, these results suggest that the highest mental effort was observed at n2, and the lowest at n0. This pattern aligns with the trends observed in offline LHIPA. While n3 shows a slight decrease compared to n2, it still indicates higher mental effort than n0.

In RIPA2, Shapiro–Wilk tests showed significant deviations from normality in most conditions (p<0.05). However, Levene’s tests indicated that the assumption of homogeneity of variances was met for RIPA2, F(3, 407)=0.584, p=0.626). To address non-normality of RIPA2, a Box–Cox transformation was applied, yielding optimized λ value of 0.308. Following transformation, normality improved considerably. The Shapiro–Wilk tests were non-significant across all the N-back levels (see Table 8), with the skewness and kurtosis values falling within acceptable ranges (|skewness|<1, |kurtosis|<1). Levene’s tests indicated homogeneity of variances across RIPA2: F=0.135, p=0.939. These results confirm that the assumption of equal variances was met after the Box–Cox transformation.

A repeated measures one-way ANOVA revealed a significant main effect of the N-back level on the transformed RIPA2, F(3, 75)=10.46, p<0.001, $\eta^{2}=0.029$, with a small effect size. Benjamini–Hochberg-adjusted pairwise post hoc comparisons showed that the values at n0 were significantly lower than those at n1 (p=0.004), n2 (p=0.003), and n3 (p=0.003). These differences were supported by strong Bayesian evidence (BF10 = 17.01 to 63.09), and effect sizes indicated small to moderate differences, with Hedges’ *g* ranging from −0.25 (n0 vs. n1) to −0.42 (n0 vs. n2) (see Table 9). Additionally, the comparison between n1 and n2 was significant (p=0.043), with moderate evidence for a real difference (BF10 = 1.98) and a small effect size (*g* = −0.25). The n1 vs. n3 was marginally significant (p=0.051), but with weak evidence for a true difference (BF10 = 1.44) and a very small effect size (*g* = −0.14).

These results suggest that n0 required the least mental effort, as indicated by the significantly lower RIPA2 values compared to n1, n2, and n3. A clear trend emerged, showing that higher N-back levels corresponded to increased mental effort, as reflected in the higher RIPA2 values. Interestingly, although n2 appeared more mentally demanding than n3, this difference was not statistically significant.

### 5.3. Discussion

In this task, we hypothesized that as the N-back levels increase, the RIPA2 values would also increase, reflecting higher mental effort. To test this, we compared RIPA2 with LHIPA and original RIPA, using only trials where the participants achieved over 80% accuracy to ensure valid data.

The results from LHIPA revealed a significant main effect of N-back level, with a small effect size, suggesting that the participants experienced increased mental effort at the n1, n2, and n3 levels compared to n0. Interestingly, the n2 level was associated with the highest mental effort among all the N-back levels, contrary to the expectation that n3 would be the most demanding. Additionally, post hoc paired comparisons using the Benjamini–Hochberg procedure showed marginal significance between n0 and n2, and between n1 and n2. No other pairwise comparisons showed significant differences.

Although the N-back task is designed so that mental demands increase linearly with the value of n, research demonstrates that this relationship plateaus or even reverses at the highest difficulty level due to participant disengagement caused by mental fatigue. Specifically, Hopstaken et al. [44] show that sustained cognitive effort leads to task disengagement: when demands exceed a personal threshold, individuals often reduce their effort or cease to fully engage, which is reflected in lower physiological markers such as pupil diameter and poorer task performance. Supporting this, Pergher et al. [45] found that measures of mental workload, such as EEG spectral power, peak at moderate difficulty levels (n2 in N-back) but do not increase further at n3; instead, higher levels of fatigue and reduced engagement explain why n2 can actually elicit greater measured effort than n3. Thus, our findings align with this literature, suggesting that n2 elicits the highest mental effort, despite n3 being designed as the most difficult level. The extreme difficulty of n3 may lead participants to disengage rather than exert additional effort.

The original RIPA, however, did not capture the expected effect of task difficulty. By definition, RIPA is expected to be positively related to mental effort, yet it showed the lowest mental effort at the n2 level and the highest at n3. While n3 showed higher mental effort than n0, both n1 and n2 had lower effort compared to n0, which is inconsistent with our expectations and with results from offline LHIPA. This suggests that the original RIPA, in its unmodified form, cannot reliably isolate mental effort, likely due to its inability to capture the relevant frequency bands. Specifically, the normalized cutoff values in the original RIPA algorithm correspond to frequencies that exceed the biologically meaningful range for pupillometry (typically below 4 Hz) [15]. Therefore, the original RIPA may have been capturing noise or measurement artifacts rather than meaningful physiological signals.

To address this, we fine-tuned the SG filters used in RIPA2 to better align with physiologically relevant frequency bands, particularly those below 4 Hz, which have been shown to correlate with cognitive load [15,32,33]. With these adjustments, the RIPA2 more accurately captured task-induced changes in mental effort during the N-back task.

As hypothesized, RIPA2 values increased with the N-back level, and the pattern of the results was consistent with LHIPA. Specifically, we observed a clear trend of increasing mental effort as the N-back level increased, with the highest RIPA2 values at n2 and a slight drop at n3. This aligns with both LHIPA and the prior literature, where n3, often considered the hardest level, is associated with task disengagement or fatigue [44,45]. A repeated-measures ANOVA revealed a significant main effect of N-back level on RIPA2, and Benjamini–Hochberg-adjusted pairwise post hoc comparisons showed that n0 values were significantly lower than those at n1, n2, and n3. In addition, comparisons between n1 and n2, and n1 and n3, were also significant, suggesting increased mental effort at higher N-back levels. Overall, these results demonstrate that RIPA2 is a sensitive and reliable measure for tracking changes in mental effort during tasks with varying difficulty levels, similar to LHIPA.

## 6. Task 2: Information Search Task

### 6.1. Method

To evaluate RIPA2’s performance in a more naturalistic setting, we employed an information-search task consisting of seven scenarios, divided into two categories: four fact-checking (FC) tasks and three decision-making (DM) tasks. The FC tasks required the participants to retrieve accurate, verifiable information from the web. For instance, the participants were asked questions such as, “*Which team won the National Basketball Association championship in 2019?*” and were expected to find and confirm the correct answer using online sources.

DM tasks, on the other hand, simulated more complex real-world scenarios where the participants evaluated multiple alternatives based on several criteria. One such task asked the participants to choose among a luxury hotel, a budget motel, and an Airbnb rental for a weekend trip, considering factors such as cost, location, amenities, and guest reviews. These tasks involved reasoning through trade-offs to make informed choices.

Example DM question: *You’re planning a weekend trip (4–6 July 2025) to New York City and need to decide where to stay. You would like to stay in a local neighborhood. You’ll need to gather information on lodging options based on price, proximity to attractions (e.g., Central Park, Times Square, Empire State Building, Brooklyn Bridge, The Metropolitan Museum of Art, and the Statue of Liberty) you are interested in visiting, and guest reviews to determine the best accommodation, considering a total lodging budget of USD 500 for the entire weekend. Which accommodation will you choose, and what factors will influence your decision?*

These information search tasks were structured using Bloom’s taxonomy [46], which categorizes cognitive processes from lower-level skills (remembering and understanding) to higher-order skills (analyzing, evaluating, and creating). Prior studies have linked larger pupil dilations to higher cognitive load during decision-making tasks [47,48,49,50,51,52].

Task difficulty in this study served as the independent variable, driven by the level of cognitive processing required. This was manipulated by comparing the DM and FC tasks, with the DM tasks designed to engage higher-order cognition, requiring the participants to integrate multiple sources, assess credibility, and justify their choices. The complexity of these tasks arise not from stimulus difficulty or memory load alone, but through the need for critical thinking and synthesis. In contrast, the FC tasks were designed to engage lower-order cognitive processes. These involved retrieving or verifying factual information and, in some cases, making basic credibility assessments.

Given the varying cognitive demands, we hypothesized that the RIPA2 values would be significantly higher during DM tasks, reflecting the greater mental effort required for complex decision making, compared to FC tasks.

All the information search tasks were conducted using the Chrome web browser embedded within iMotions, with DuckDuckGo set as the default search engine. DuckDuckGo was selected because, at the time of this study, it allowed disabling generative AI features, an option not available with popular search engines such as Google. To maintain consistency and minimize the influence of artificial intelligence tools, all AI-assisted search features were turned off. The participants were allowed to open multiple browser tabs and were free to navigate any websites, with the exception of generative AI platforms, which were explicitly restricted.

The DM tasks were capped at a maximum duration of 15 min, while FC tasks had no time limit. In DM tasks, the participants either made a decision based on the information they had gathered or reached the 15-minute limit. At that point, they closed the browser and entered their final decision into a text box. They were also prompted to provide a brief verbal explanation of the key factors that influenced their choice for the DM task. In contrast, the FC tasks were typically completed more quickly, on average, within 2 min. The participants simply submitted their final answer after completing the search and closing the browser.

### 6.2. Results

Similar to the N-back task, we computed the LHIPA, original RIPA, and RIPA2 values for each information search task, which were used as the dependent variables in our analysis. To ensure appropriate statistical analysis, we first assessed the normality of LHIPA, RIPA, and RIPA2. All three measures showed significant deviations from normality across several task conditions (p<0.05 in most cases). As a result, we applied a Box–Cox transformation to normalize the data distributions, and then conducted repeated measures ANOVA for comprehensive statistical evaluation.

#### 6.2.1. LHIPA

**Figure 7:**
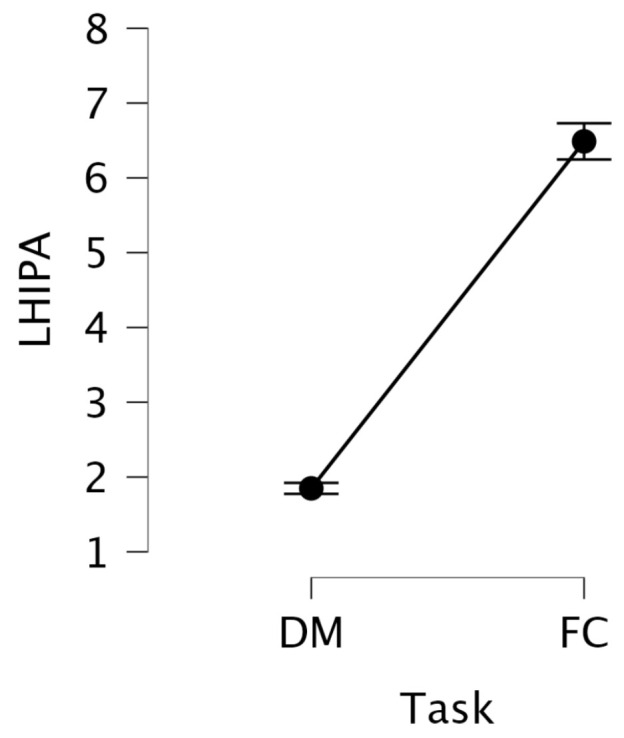
Mean LHIPA values with standard error bars across DM and FC information search tasks.

We observed that LHIPA was substantially higher during the FC task (M = 6.49, SD = 2.63) compared to the DM task (M = 1.85, SD = 0.70) (see Figure 7). Since LHIPA is inversely related to cognitive load, this suggests that the participants experienced lower mental effort during the FC task compared to the DM task.

Prior to conducting the statistical analysis, through a Levene’s test, we observed a significant violation of the homogeneity of variances assumption for LHIPA across DM and FC tasks, F(1, 206)=102.73, p<0.001. Additionally, the Shapiro–Wilk test revealed that the data were not normally distributed for either the DM (W=0.873, p<0.001) or the FC task (W=0.927, p<0.001).

To address violations of normality and homogeneity of variance in the raw LHIPA data, a Box–Cox transformation was applied with a λ of 0.0907. This transformation improved the distributional properties of the data. Post-transformation, the Shapiro–Wilk test indicated no significant departures from normality in either the DM task (W=0.967, p=0.469) or the FC task (W=0.956, p=0.248). Similarly, Levene’s test showed no violation of homogeneity of variances across tasks (F(1, 29)=0.0127, p=0.9105), supporting the use of parametric analyses.

A repeated-measures ANOVA on the transformed LHIPA revealed a highly significant main effect of task, F(1, 29)=458.076, p<0.0001, with a large effect size ($\eta^{2}=0.827$). This confirms that LHIPA was significantly greater during the FC task compared to the DM task, even after addressing distributional assumptions. Given that higher LHIPA values reflect lower mental effort, this indicates that participants exhibited increased mental effort during the DM task than during the FC task.

#### 6.2.2. RIPA

**Figure 8:**
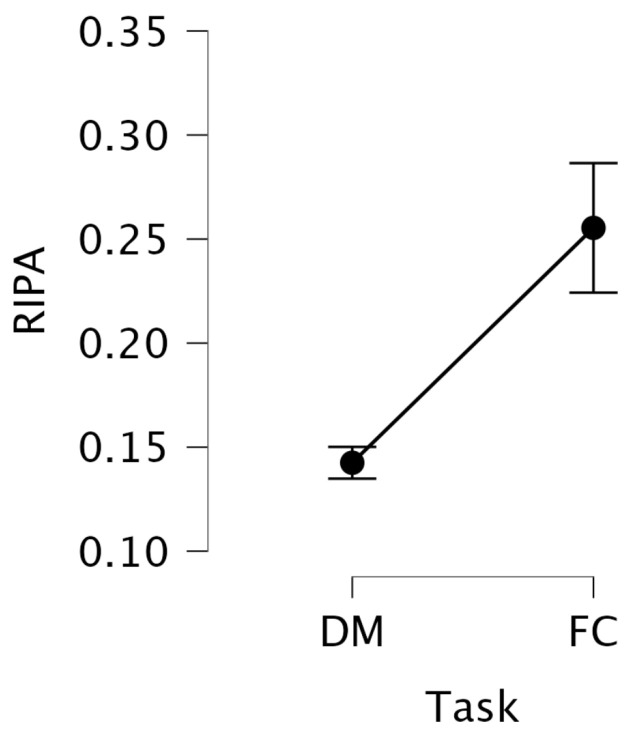
Mean RIPA values with standard error bars across DM and FC, information search tasks.

Descriptive statistics of RIPA show an increase from the DM task (M = 0.142, SD = 0.072) to the FC task (M = 0.255, SD = 0.338) (see Figure 8). Since RIPA increases with cognitive load, this suggests that the FC task was associated with higher mental effort. However, this finding is inconsistent with offline LHIPA results, where we observed that participants exhibited greater mental effort during DM task.

Prior to conducting statistical analysis, we assessed the data distribution. Levene’s test indicated a significant violation of the homogeneity of variances assumption across tasks, F(1, 206)=76.89, p<0.001. Moreover, the Shapiro–Wilk test revealed that RIPA was not normally distributed during either the DM (W=0.959, p=0.006) or FC task (W=0.589, p<0.001).

The Box–Cox transformation of RIPA (λ=−0.4567) improved data normality (Shapiro–Wilk of DM: W=0.944, p=0.114; FC: W=0.967, p=0.468) and homogeneity of variances (Levene’s test F(1, 29)=0.288, p=0.594). However, a repeated-measures ANOVA on the transformed RIPA data revealed no significant main effect of task, F(1, 29)=0.0236, p=0.8787.

#### 6.2.3. RIPA2

**Figure 9:**
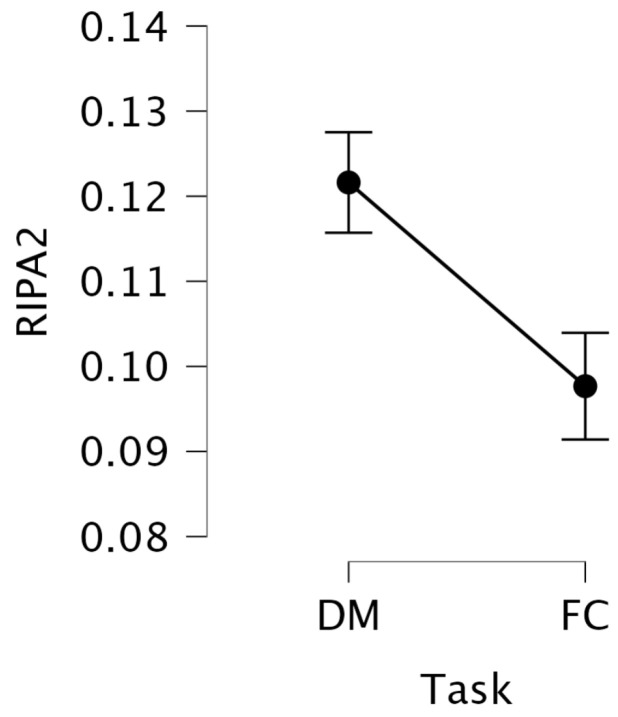
Mean RIPA2 values with standard error bars for DM and FC tasks.

For RIPA2, the mean value was 0.122 (SD = 0.056) during the DM task and 0.098 (SD = 0.067) during the FC task (see Figure 9). Since RIPA2 is designed to increase with mental effort, this suggests that the participants experienced lower mental effort during the FC task compared to the DM task, consistent with the trend observed in LHIPA.

The Shapiro–Wilk tests indicated significant deviations from normality for RIPA2 in both the DM (W=0.958, p=0.006) and FC (W=0.838, p<0.001) tasks, with the FC task showing notably higher skewness (1.903) and kurtosis (4.839), suggesting a more non-normal distribution (see Table 10). Levene’s test confirmed that variances were homogeneous between conditions (F(1, 203)=0.446, p=0.505).

To meet the assumptions of repeated-measures ANOVA, RIPA2 was transformed using the Box–Cox method (λ=0.3181). After transformation, RIPA2 approximated normality across tasks, with the Shapiro–Wilk tests yielding non-significant results for both the DM task (W=0.987, p=0.968) and FC task (W=0.982, p=0.883). The skewness values were −0.064 for the DM task and −0.369 for the FC task, both within acceptable bounds. Similarly, the kurtosis values were −0.093 for the DM task and −0.306 for the FC task, indicating normality. Homogeneity of variances was confirmed with Levene’s test, which showed no significant differences between groups (F=0.091, p=0.764).

A repeated-measures ANOVA on the transformed RIPA2 revealed a significant main effect of task, F(1, 29)=31.86, p<0.001, $\eta^{2}=0.083$, indicating greater mental effort during the DM task compared to the FC task, with a medium-to-large effect size. This result supports our hypothesis and is consistent with findings from the offline LHIPA analysis.

### 6.3. Discussion

To assess RIPA2 in a naturalistic setting, we conducted an information search task with both the FC and DM tasks. The FC task was simpler, while the DM task required more complex decision-making. We hypothesized that the RIPA2 values would be higher during the DM tasks due to the greater cognitive load involved in critical thinking and judgment.

Our findings were consistent with this hypothesis. Both the LHIPA and RIPA2 results revealed that the participants exhibited greater mental effort during the DM task compared to the FC task. A repeated-measures ANOVA on LHIPA showed a highly significant main effect of task, with a large effect size. Similarly, RIPA2 also exhibited a significant main effect of task, with a medium-to-large effect size. These results support our expectation that DM tasks, which involve more complex cognitive processing, such as integrating multiple sources of information, evaluating credibility, and making judgment calls, demand more mental effort than the simpler FC tasks, which primarily involve factual recall or basic validation. Specifically, the lower LHIPA values and higher RIPA2 values during the DM task reflect increased mental effort, aligning with our hypothesis.

Interestingly, the original RIPA did not capture these task-related differences in mental effort. This highlights one of the key strengths of RIPA2, which was able to track mental effort fluctuations in a way that aligned with offline LHIPA results. By overcoming the limitations of the original RIPA, RIPA2 effectively captured task-related fluctuations in mental effort. Its ability to detect these differences suggests that RIPA2 is more sensitive to the varying cognitive demands of different tasks. This provides strong evidence for the utility of RIPA2 as a more accurate and reliable measure of mental effort in near-real-time, applicable in both structured laboratory settings and naturalistic tasks.

## 7. General Discussion and Future Directions

This study aimed to enhance a real-time pupillometric approach for measuring mental effort by refining the original RIPA method [11] and validating the enhanced RIPA2 across both structured and naturalistic cognitive tasks. The results demonstrate that RIPA2 reliably captures fluctuations in mental effort in response to varying task demands, addressing the key limitations of the original approach.

The RIPA2 method, by making targeted adjustments to SG filter parameters, particularly within biologically relevant frequency bands of pupil diameter fluctuations, demonstrated its ability to detect meaningful changes in mental effort. This refinement allowed RIPA2 to align more closely with offline measures, similar to LHIPA and theoretical expectations. For example, in the N-back task, RIPA2 showed a peak in mental effort at the n2 level rather than at the highest difficulty level (n3), reflecting a pattern of task disengagement under excessive cognitive demand, an effect also captured by offline LHIPA.

Beyond structured, artificial cognitive tasks, RIPA2 effectively distinguished between high and low mental effort in a more naturalistic information search task, specifically between FC and DM scenarios. This demonstrates RIPA2’s robustness in tracking mental effort in complex, real-world contexts involving information integration, credibility assessment, and judgment. Its performance in this setting suggests strong potential for broader application in monitoring mental effort outside the laboratory, such as in educational settings, medical environments, workplace environments, or adaptive user interfaces.

### Limitations and Future Directions

Although RIPA2 functions as a real-time pupillometric index of mental effort, it is not without certain limitations. First, RIPA2 was validated on only two laboratory-based tasks with a homogeneous sample of university students, limiting its generalizability. To further evaluate RIPA2’s generalizability, future studies should validate the method across a wider range of cognitive tasks and populations. Expanding RIPA2 to include tasks targeting other cognitive domains, such as attention, problem-solving, and creativity, will help us assess its versatility. In addition, applying RIPA2 in real-world contexts (e.g., classrooms, workplaces, or driving simulations) will offer insights into its effectiveness across diverse environments. Another key area for improvement is participant diversity, as the current study focused solely on university students. Future research should include broader populations, such as children, older adults, and individuals with cognitive impairments, to assess RIPA2’s adaptability across different age groups and cognitive profiles.

Second, the proposed method relies on high-quality pupillometry data collected at a 300 Hz sampling frequency. Future evaluations should consider eye-trackers with lower sampling rates to assess performance across a broader range of devices.

While the observed effects were generally small to moderate, this suggests that further refinement is needed to enhance the robustness and applicability of the proposed approach.

RIPA2 currently treats blinks as artifacts, using a gap removal approach that excludes affected segments from the data. However, previous work has shown that pupil dilation and blinks provide complementary, mutually exclusive indices of information processing: blinks often occur during early sensory or transient processing, whereas pupil dilation reflects sustained cognitive effort [53]. This suggests that incorporating blink frequency and timing alongside pupil diameter could enable richer, multimodal assessments of cognitive load in future studies, particularly in naturalistic or high-stress settings. Moving forward, we aim to implement real-time blink gap interpolation to preserve more of the pupil signal and improve temporal continuity and integrate blinks into RIPA2. These enhancements could further increase the accuracy and reliability of RIPA2 in real-time applications.

Lastly, RIPA2 focuses exclusively on pupil diameter as the physiological measure, without integrating multimodal data (e.g., heart rate, galvanic skin response, EEG, blinks, emotional variables, and functional near-infrared spectroscopy (fNIRS)) which could provide a more comprehensive understanding of cognitive load. In the future, we plan to compare RIPA2 with other physiological measures to assess its performance relative to established multimodal indicators of cognitive load. Demonstrating convergence between RIPA2 and these measures would further validate its effectiveness and position it as a robust, practical tool for assessing mental effort in both research and applied settings.

## 8. Conclusions

We introduced RIPA2, a refined and validated pupillometric index of mental effort, specifically designed for real-time performance. The key innovation in RIPA2 is the adjustment of Savitzky–Golay filter parameters to isolate frequency components relevant to cognitive load. Unlike the original RIPA, which prioritized responsiveness over spectral precision, RIPA2 aligns Savitzky–Golay filter frequency responses with physiologically meaningful bands: the first filter targets very-low-frequency fluctuations (≤0.29 Hz), and the second captures low-frequency fluctuations (≤4 Hz), reflecting task-evoked cognitive processes.

RIPA2 was evaluated using two tasks: the N-back task, a structured cognitive benchmark, and an information-search task involving fact-checking and decision-making, a more naturalistic scenario. Across both tasks, RIPA2 demonstrated greater sensitivity and reliability than the original RIPA, with the results consistent with offline LHIPA measure. These findings indicate that RIPA2 can reliably track cognitive load in both controlled and complex, real-world tasks requiring information integration, judgment, and decision-making. Consequently, RIPA2 holds potential for broader applications, including adaptive learning systems, dynamic user interfaces, medical and workplace environments, driving safety monitoring, and immersive VR/AR environments.

## Figures and Tables

**Table 1:** Descriptive statistics for LHIPA values across N-back levels.

	LHIPA			
	n0	n1	n2	n3
Mean	5.063	5.002	4.844	4.932
Std. Deviation	0.692	0.764	0.865	0.834
Skewness	−0.422	−0.594	−0.769	−0.419
Kurtosis	−0.530	−0.279	0.195	−0.790
Shapiro–Wilk	0.968	0.958	0.951	0.956
*p*-value (SW)	0.010	0.001	<0.001	0.005

**Table 2:** Normality and homogeneity statistics for Box–Cox-transformed LHIPA Across N-back levels.

Statistic	n0	n1	n2	n3
Skewness	0.006	−0.073	−0.216	0.077
Kurtosis	−1.226	−1.132	−0.822	−1.076
Shapiro–Wilk (W)	0.942	0.950	0.948	0.951
*p*-value (SW)	0.148	0.234	0.211	0.240

**Table 3:** Post hoc pairwise comparisons of Box–Cox-transformed LHIPA across N-back levels with Benjamini–Hochberg correction.

Contrast	*t*	df	*p*-Uncorrected	*p*-Corrected	BF_10_	Hedges’ *g*
n0 vs. n1	1.29	25	0.210	0.253	0.43	0.08
n0 vs. n2	2.22	25	0.036	0.069	1.66	0.29
n0 vs. n3	2.66	25	0.014	0.054	3.65	0.31
n1 vs. n2	2.10	25	0.046	0.069	1.34	0.20
n1 vs. n3	2.53	25	0.018	0.054	2.90	0.23
n2 vs. n3	0.58	25	0.568	0.568	0.24	0.04

**Table 4:** Descriptive statistics for RIPA values across N-back levels.

	RIPA			
	n0	n1	n2	n3
Mean	0.227	0.188	0.149	0.280
Std. Deviation	0.339	0.300	0.241	0.386
Skewness	1.758	2.285	3.123	1.307
Kurtosis	1.331	3.487	8.465	−0.210
Shapiro–Wilk	0.581	0.505	0.453	0.598
*p*-value (SW)	<0.001	<0.001	<0.001	<0.001

**Table 5:** Distributional statistics for Box–Cox-transformed RIPA across N-back levels.

	n0	n1	n2	n3
Skewness	0.128	0.417	0.084	−0.005
Kurtosis	0.427	0.504	−0.212	−0.857
Shapiro–Wilk *W*	0.968	0.980	0.979	0.959
*p*-value (SW)	0.579	0.879	0.869	0.384

**Table 6:** Post hoc pairwise comparisons for RIPA across N-back levels (Box–Cox-transformed) with Benjamini–Hochberg procedure.

Contrast	*t*	df	*p*-Uncorrected	*p*-Corrected	BF_10_	Hedges’ *g*
n0 vs. n1	−0.23	25	0.818	0.841	0.21	−0.06
n0 vs. n2	0.20	25	0.841	0.841	0.21	0.04
n0 vs. n3	−1.19	25	0.244	0.733	0.39	−0.29
n1 vs. n2	0.46	25	0.652	0.841	0.23	0.11
n1 vs. n3	−0.81	25	0.426	0.841	0.28	−0.25
n2 vs. n3	−1.59	25	0.124	0.733	0.63	−0.40

**Table 7:** Descriptive statistics for RIPA2 values across N-back levels.

	RIPA2			
	n0	n1	n2	n3
Mean	0.159	0.180	0.206	0.178
Std. Deviation	0.103	0.102	0.104	0.096
Skewness	1.363	0.896	0.498	0.748
Kurtosis	1.725	0.426	−0.527	0.506
Shapiro–Wilk (W)	0.879	0.936	0.963	0.953
*p*-value (SW)	<0.001	<0.001	0.006	0.003

**Table 8:** Descriptive statistics and normality tests for Box–Cox-transformed data.

Statistic	RIPA2			
	n0	n1	n2	n3
Skewness	0.501	−0.228	−0.563	−0.402
Kurtosis	0.274	−0.151	0.057	−0.453
Shapiro–Wilk *W*	0.967	0.978	0.968	0.964
Shapiro–Wilk *p*	0.537	0.829	0.559	0.482

**Table 9:** Post hoc pairwise comparisons for RIPA2 across N-back levels (Box–Cox-transformed) with Benjamini–Hochberg procedure.

Contrast	*t*	df	*p*-Uncorrected	*p*-Corrected	BF_10_	Hedges’ *g*
n0 vs. n1	−3.41	25	0.002	0.004	17.01	−0.25
n0 vs. n2	−3.63	25	0.001	0.004	27.63	−0.42
n0 vs. n3	−3.99	25	0.0005	0.003	63.09	−0.39
n1 vs. n2	−2.32	25	0.029	0.043	1.98	−0.18
n1 vs. n3	−2.14	25	0.043	0.051	1.44	−0.14
n2 vs. n3	0.65	25	0.521	0.521	0.25	0.04

**Table 10:** Descriptive statistics for RIPA2 values across DM and FC tasks.

	RIPA2	
	DM	FC
Mean	0.122	0.098
Std. Deviation	0.056	0.067
Skewness	0.812	1.903
Kurtosis	0.749	4.839
Shapiro–Wilk (W)	0.958	0.838
*p*-value (SW)	0.006	<0.001

## Data Availability

The original contributions presented in this study are included in the article. Further inquiries can be directed to the corresponding author.
